# Exercise following joint distraction inhibits muscle wasting and delays the progression of post-traumatic osteoarthritis in rabbits by activating PGC-1α in skeletal muscle

**DOI:** 10.1186/s13018-024-04803-y

**Published:** 2024-05-31

**Authors:** Xinghui Liu, Rong Chen, Zhenfei Song, Zhibo Sun

**Affiliations:** 1 School of Basic Medical Sciences, Hubei University of Arts and Science, Xiangyang, Hubei 441000 China; 2 Department of Traumatic Orthopedics, Xiangyang Hospital of Traditional Chinese Medicine (Xiangyang Institute of Traditional Chinese Medicine), No. 24 Changzheng Road, Xiangyang, Hubei 441001 China

**Keywords:** Post-traumatic osteoarthritis, Joint distraction, Exercise, PGC-1α, Muscle wasting

## Abstract

**Objective:**

Muscle wasting frequently occurs following joint trauma. Previous research has demonstrated that joint distraction in combination with treadmill exercise (TRE) can mitigate intra-articular inflammation and cartilage damage, consequently delaying the advancement of post-traumatic osteoarthritis (PTOA). However, the precise mechanism underlying this phenomenon remains unclear. Hence, the purpose of this study was to examine whether the mechanism by which TRE following joint distraction delays the progression of PTOA involves the activation of peroxisome proliferator-activated receptor gamma coactivator 1-alpha (PGC-1α), as well as its impact on muscle wasting.

**Methods:**

Quadriceps samples were collected from patients with osteoarthritis (OA) and normal patients with distal femoral fractures, and the expression of PGC-1α was measured. The hinged external fixator was implanted in the rabbit PTOA model. One week after surgery, a PGC-1α agonist or inhibitor was administered for 4 weeks prior to TRE. Western blot analysis was performed to detect the expression of PGC-1α and Muscle atrophy gene 1 (Atrogin-1). We employed the enzyme-linked immunosorbent assay (ELISA) technique to examine pro-inflammatory factors. Additionally, we utilized quantitative real-time polymerase chain reaction (qRT-PCR) to analyze genes associated with cartilage regeneration. Synovial inflammation and cartilage damage were evaluated through hematoxylin-eosin staining. Furthermore, we employed Masson’s trichrome staining and Alcian blue staining to analyze cartilage damage.

**Results:**

The decreased expression of PGC-1α in skeletal muscle in patients with OA is correlated with the severity of OA. In the rabbit PTOA model, TRE following joint distraction inhibited the expressions of muscle wasting genes, including Atrogin-1 and muscle ring finger 1 (MuRF1), as well as inflammatory factors such as interleukin-1β (IL-1β) and tumor necrosis factor-α (TNF-α) in skeletal muscle, potentially through the activation of PGC-1α. Concurrently, the production of IL-1β, IL-6, TNF-α, nitric oxide (NO), and malondialdehyde (MDA) in the synovial fluid was down-regulated, while the expression of type II collagen (Col2a1), Aggrecan (AGN), SRY-box 9 (SOX9) in the cartilage, and superoxide dismutase (SOD) in the synovial fluid was up-regulated. Additionally, histological staining results demonstrated that TRE after joint distraction reduced cartilage degeneration, leading to a significant decrease in OARSI scores.TRE following joint distraction could activate PGC-1α, inhibit Atrogin-1 expression in skeletal muscle, and reduce C-telopeptides of type II collagen (CTX-II) in the blood compared to joint distraction alone.

**Conclusion:**

Following joint distraction, TRE might promote the activation of PGC-1α in skeletal muscle during PTOA progression to exert anti-inflammatory effects in skeletal muscle and joint cavity, thereby inhibiting muscle wasting and promoting cartilage regeneration, making it a potential therapeutic intervention for treating PTOA.

## Background

Post-traumatic osteoarthritis (PTOA) is a form of osteoarthritis (OA) that arises from joint trauma, such as intra-articular fractures, cruciate ligament tears, and meniscal injuries, which seriously affects the quality of life of patients and increases the medical burden [1]. In addition to factors like joint instability and uneven surfaces, chronic inflammation within the joints plays a crucial role in the progression of PTOA [2]. Specifically, interleukin-1β (IL-1β) is a highly pro-inflammatory cytokine that triggers the release of various other pro-inflammatory cytokines and catabolic factors, including IL-6, tumor necrosis factor-α (TNF-α), and matrix metalloproteinases (MMPs), disrupting the balance between extracellular matrix (ECM) anabolism and catabolism [3]. Inflammation and mechanical stress responses following trauma can lead to mitochondrial dysfunction, exacerbating cartilage degeneration. However, enhancing mitochondrial function may potentially prevent or treat PTOA by suppressing intra-articular inflammation and slowing cartilage degeneration [4]. Since patients with PTOA are typically younger than those with primary OA, joint replacement surgery is not the preferred approach. Instead, joint distraction is recommended as a joint-preserving surgical treatment technique [5]. Moreover, joint distraction has the potential to delay the wear and tear of cartilage [6], decrease the inflammatory response [7], and enhance cartilage repair [8], thereby alleviating joint pain and improving joint function of patients. A member of our research group discovered the combination of joint distraction and treadmill exercise (TRE) may have beneficial effects in inhibiting intra-articular inflammation and promoting cartilage regeneration and repair [9]. However, further investigation is required to understand the specific mechanisms involved.

Muscle wasting frequently occurs following joint trauma and is strongly linked to the progression of OA. It plays a crucial role in the development of OA by exacerbating joint instability and consequent joint wear [10]. Moreover, chronic inflammation in OA patients is closely associated with muscle wasting [11]. The reduced joint movement in OA, caused by pain during exercise, leads to muscle wasting [12]. However, exercise has been shown to enhance muscle mass and decrease levels of inflammatory markers, such as TNF-α, C-reactive protein (CRP), and IL-6, in patients with rheumatoid arthritis (RA) [13]. Additionally, exercise can improve mitochondrial function and activate peroxisome proliferator-activated receptor gamma coactivator 1-alpha (PGC-1α) in skeletal muscle, which is a key regulator of mitochondrial biogenesis and skeletal muscle metabolism [14]. Aerobic and resistance exercises elevate the transcription and translation levels of PGC-1α in skeletal muscles, enhancing mitochondrial function while suppressing inflammation and oxidative stress [15]. The absence of PGC-1α in skeletal muscle leads to decreased muscle function and increased inflammation [15]. Moreover, skeletal muscle inflammation and wasting are closely associated with OA progression. Exercise enhances the anti-inflammatory effect of skeletal muscles and reduces the expression of pro-inflammatory factors in RA patients [13]. Furthermore, muscle wasting may result in joint instability, leading to mechanical stress-induced inflammation [10]. Hence, exercise promotes PGC-1α in skeletal muscle, which may improve muscle inflammation and wasting, thereby reducing joint inflammation. However, in cases of joint instability, even moderate exercise can worsen the loss of proteoglycans and contribute to the degeneration of articular cartilage [16]. Therefore, it is crucial to stabilize the knee joint using a hinged external fixator before engaging in exercise [9]. However, it is still unclear whether exercise-induced activation of PGC-1α in skeletal muscle of an unstable joint is dependent on joint distraction.

Therefore, the hypothesis of this study is that in a rabbit joint model with instability, TRE following joint distraction improves muscle wasting, inhibits intra-articular inflammation, and promotes cartilage regeneration by activating skeletal muscle PGC-1α, thereby delaying the progression of PTOA. As previously reported [9], a model of knee instability in rabbits was constructed using the anterior cruciate ligament transection (ACLT) and fixed with a hinged external fixator, with TRE performed 1 week postoperatively. Furthermore, the PGC-1α interfering agent was used before TRE to investigate markers of muscle wasting, intra-articular inflammation, and cartilage regeneration. Our aim is to contribute new ideas for the prevention and treatment of PTOA.

## Materials and methods

### Collection of human quadriceps tissue samples

Specimens of the vastus intermedius muscle from patients with OA who underwent total knee replacement at the Orthopedic Center of Xiangyang Hospital of Traditional Chinese Medicine were collected. Normal human quadriceps specimens were obtained from patients with distal femoral fractures. The collection and use of human biological materials were approved by the Ethics Committee of Xiangyang Hospital of Traditional Chinese Medicine (NO.2023-02). Written informed consent was obtained from all subjects prior to surgery.

### Construction of PTOA rabbit model and experimental design

**Fig. 1:**
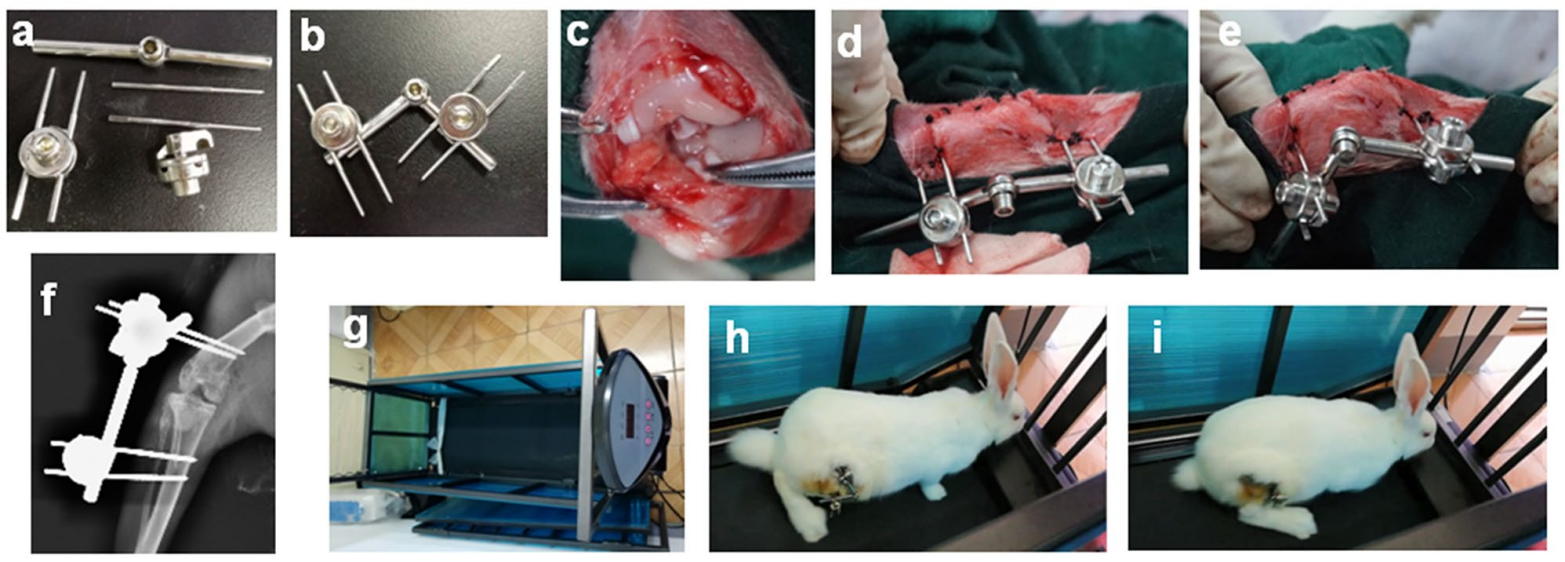
Installation of hinged external fixators after PTOA model establishment, and treadmill training for rabbits. **a**-**b** The disassembly and installation of the hinged external fixator **c** PTOA model establishment **d-e** Joint extension and flexion following joint distraction **f** The X-ray examination image following the installation of hinged external fixators **g** The animal treadmill **h-i** Rabbits exercising on the treadmill

All experimental designs were conducted in accordance with the guidelines set by the Cornell Institutional Animal Care and Use Committee of Hubei University of Medicine (NO.2018 − 100), following the Animal Research: Reporting of In Vivo Experiments guideline. Male New Zealand rabbits, aged 5–6 months and weighing 2.8–3.1 kg, were obtained from the Animal Experimental Center of Hubei University of Medicine. Prior to the surgery, all rabbits underwent low-speed treadmill exercise (13 m/minute for 10 min) for 5 days. To induce PTOA, the rabbits were first anesthetized with a 3% pentobarbital injection (1 ml/kg) via the auricular vein. Under aseptic conditions, a 2–3 cm anterior medial curve incision was made on the right knee, and the anterior cruciate ligament was excised for 0.5 cm. The success of the operation was confirmed using the front drawer test. The rabbits in the sham group underwent only an incision without ligament transection. Joint distraction was achieved using a hinged external fixator, as previously described [9]. The figures illustrate the disassembly and installation of the hinged external fixator (Fig. 1a, b), establishment of the PTOA model (Fig. 1c), joint extension and flexion after joint distraction (Fig. 1d, e), postoperative X-ray images (Fig. 1f), the animal treadmill (Fig. 1g), and rabbits exercising on the treadmill (Fig. 1h, i). Intramuscular injection of penicillin (400,000 units/kg) was administered 30 min before the surgery and continued for 3 days post-surgery. One week after the surgery, all rabbits in the TRE group began treadmill training at a speed of 17 m/min for 4 weeks (15 min per day, 5 days per week) [16]. In the TRE group, 30 rabbits were randomly assigned to 5 groups, with 6 rabbits in each group: Sham group, PTOA group, PTOA + ZLN005 group (ZLN005 administered orally at a dosage of 15 mg/kg per day for 4 weeks), PTOA + D group (joint distraction using a hinged external fixator), and PTOA + D + SR-18,292 group (in addition to joint distraction, SR-18,292 injected intraperitoneally at a dosage of 45 mg/kg per day for 4 weeks). In the No TRE group, 12 rabbits were randomly assigned to 2 groups, with 6 rabbits in each group: PTOA + D group (No TRE), and PTOA + D + ZLN005 group (No TRE). The dosage of ZLN005 and SR-18,292 was determined based on the provided instructions.

### Western blot (WB)

The radioimmunoprecipitation assay (RIPA) lysis buffer was used to extract the total proteins from vastus intermedius muscle tissues. Protein concentrations were determined using BCA protein concentration determination method (Beyotime, China). Subsequently, 30 µg of total proteins were loaded for each group and separated by 10% SDS-PAGE, followed by transfer onto a PVDF membrane. After blocking with 5% non-fat milk at 25 °C for 1 h, the membranes were incubated overnight at 4 °C with primary antibodies against PGC-1α (1:1000), Atrogin-1 (1:1000), and GAPDH (1:5000) from HuaAn Biotechnology (China). The next day, the membranes were washed three times with TBST and incubated with an HRP-conjugated secondary antibody (1:10000) at 25 °C for 1 h. Finally, the protein bands were visualized using the ECL reagent and an Imaging System from Bio-Rad (USA).

### Quantitative real-time PCR (qRT-PCR)

The total RNA was extracted from the vastus intermedius muscle and medial condyle cartilage of the femur using the TRIzol reagent (Invitrogen, Carlsbad, CA, USA) according to the manufacturer’s instructions. Subsequently, the extracted total RNA was transcribed into first-strand cDNA. The *PGC-1α, Muscle atrophy gene 1(Atrogin-1), muscle ring finger 1(MuRF1), IL-1β, TNF-a, type II collagen (Col2a1), Aggrecan (AGN) and SRY-box 9 (SOX9)* genes (Table 1) were amplified with specific primers in SYBR Green/ROX qPCR Master Mix. GAPDH was used as an internal loading control. The reactions were performed in a CFX96 qRT-PCR detection system (Bio-Rad, USA). The specific amplification of the gene of interest was validated using the melting curve. The relative gene expression was calculated from the CT values of amplification curve using 2^−ΔΔCт^ method.

**Table 1:** Primers used for assessing the gene expressions using the qRT-PCR

Gene	Forward (sequence 5′- 3′)	Reverse (sequence 5′- 3′)
Homo *PGC-1α*	CCTGCATGAGTGTGTGCTCT	GCAAAGAGGCTGGTCTTCAC
Rabbit *Atrogin-1*	TACTGCACTTTGGGGGAAGC	ATCAGTTCCAACAGCCGGAC
Rabbit *MuRF1*	CACCTTCCTCATGAGTGCCA	TCTGTCCCAAAGTCGATGGC
Rabbit *IL-1β*	GCC GAT GGT CCC AAT TAC AT	ACA AGA CCT GCC GGA AGC T
Rabbit *TNF-α*	TCT AGT CAA CCC TGT GGC CC	GCC CGA GAA GCT GAT CTA AG
Rabbit *Col2a1*	AGTCTTGCCCCACTTACC	TCCCAGAACATCACCTACCA
Rabbit *AGN*	CCACAGACCCTAAGCCTTCT	CCACAGACCCTAAGCCTTCT
Rabbit SOX-9	GGTGAAGGTGGAGTAGAGGCT	GAACGCACATCAAGACGGA
Rabbit *GAPDH*	GGAGGCAGGGATGATGTTCT	TGTTTGTGATGGGCGTGAA

### Measurement of IL-1β, IL-6, TNF-α, NO, MAD, SOD and CTX-II

We collected joint cavity effusion and blood samples from each rabbit to measure IL-1β, IL-6, TNF-α, and C-telopeptides of type II collagen (CTX II) using Enzyme-linked immunosorbent assay (ELISA), following the instructions provided by the manufacturer (SinoBestBio, China). We measured the levels of nitric oxide (NO), malondialdehyde (MDA), and superoxide dismutase (SOD) in the sample supernatant using the respective assay kits, as per the instructions provided by the manufacturer (SinoBestBio, China).

### Histological analysis

The medial condyle of the femur in the knee joint was fixed in a 4% paraformaldehyde solution, decalcified, embedded in paraffin, and then sectioned into 5 μm thick paraffin tissue sections. Follow the manufacturer’s instructions to assess the morphology of chondrocytes and the presence of synovial inflammation using hematoxylin-eosin staining (HE), determine collagen alignment using Masson’s trichrome staining (Masson), and analyze proteoglycan distribution using Alcian blue staining (Alcian blue). The slides were analyzed using an optical microscope that was equipped with a digital CCD camera manufactured by Olympus in Japan. The severity of cartilage damage and inflammation of the synovial tissue in the medial compartment of the joint cavity were assessed using the Osteoarthritis Research Society International (OARSI) scoring system, following the previously described method [16, 17].

### Statistical analysis

All data were analyzed using statistical software SPSS version 25.0 (IBM Corporation, USA). The data were presented as mean ± standard derivation. The χ^2^ test was used to compare gender and underlying diseases between the two patient groups, while the t-test was employed to compare ages and levels of PGC-1α. Synovitis scores and cartilage OARSI scores were analyzed using one-way ANOVA, while other indicators were analyzed using two-way ANOVA. If the assumption of homogeneity of variance was met for the data, the Bonferroni method was used for the multiple comparisons. If the assumption of homogeneity of variance was not met for the data, the Games-Howell method was used alternatively. Correlations between the mRNA expression levels of PGC-1α in human OA muscle samples and K-L grade were measured using Spearman’s rank correlation coefficient ρ. To assess the correlation between PGC-1α and Atrogin-1, IL-1β in skeletal muscle, IL-1β in the joint cavity and Col2a1 in the cartilage, linear regression analysis was performed and the coefficients of determination R^2^ and the *P* values were calculated. Differences were considered as significant when *P* value < 0.05.

## Results

### Decreased expression of PGC-1α in skeletal muscle in patients correlated with OA severity

**Fig. 2:**
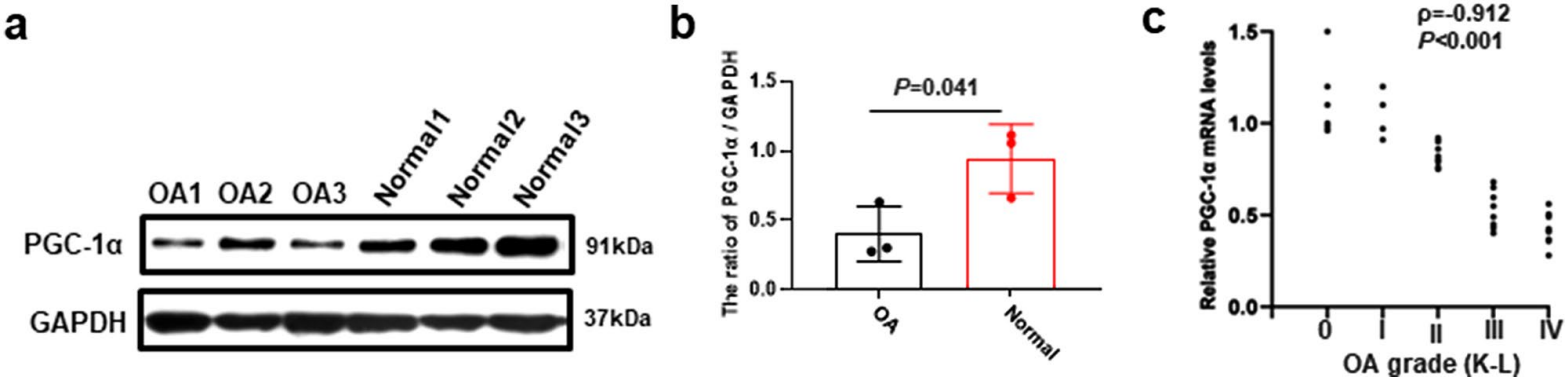
Decreased expression of PGC-1α in skeletal muscle in patients correlated with OA severity **a**-**b** Western blot analysis was performed to detect and quantify the expression of PGC-1α in the quadriceps muscle. **c** The Spearman’s rank correlation was calculated between the mRNA expression of PGC-1α and the K-L grade

No significant differences were observed in baseline characteristics, including age, gender, and underlying disease, between the two groups (Table 2). The expression of PGC-1α in the quadriceps of patients was detected using both WB and qRT-PCR. Figure 2a and b demonstrated a decrease in protein expression of PGC-1α in patients with OA. Additionally, the mRNA expression levels of PGC-1α showed a negative correlation with the K-L grade of OA severity, as indicated by Spearman’s rank correlation coefficient (Fig. 2c).

**Table 2:** Comparison of general demographics (mean ± SD; *n*, %)

	OA group (*n* = 3)	Normal group (*n* = 3)	t/χ2	*P*
Gender			0	1
Male	1(33.33)	1(33.33)		
Female	2(66.67)	2(66.67)		
Age(years)	68.33 ± 5.51	67.33 ± 8.74	0.168	0.875
Underlying disease			0	1
No	1(33.33)	1(33.33)		
Diabetes	1(33.33)	1 (33.33)		
Cardiovascular disease	1(33.33)	1(33.33)		

### TRE following joint distraction inhibited muscle wasting and inflammation by activating PGC-1α in the PTOA model

**Fig. 3:**
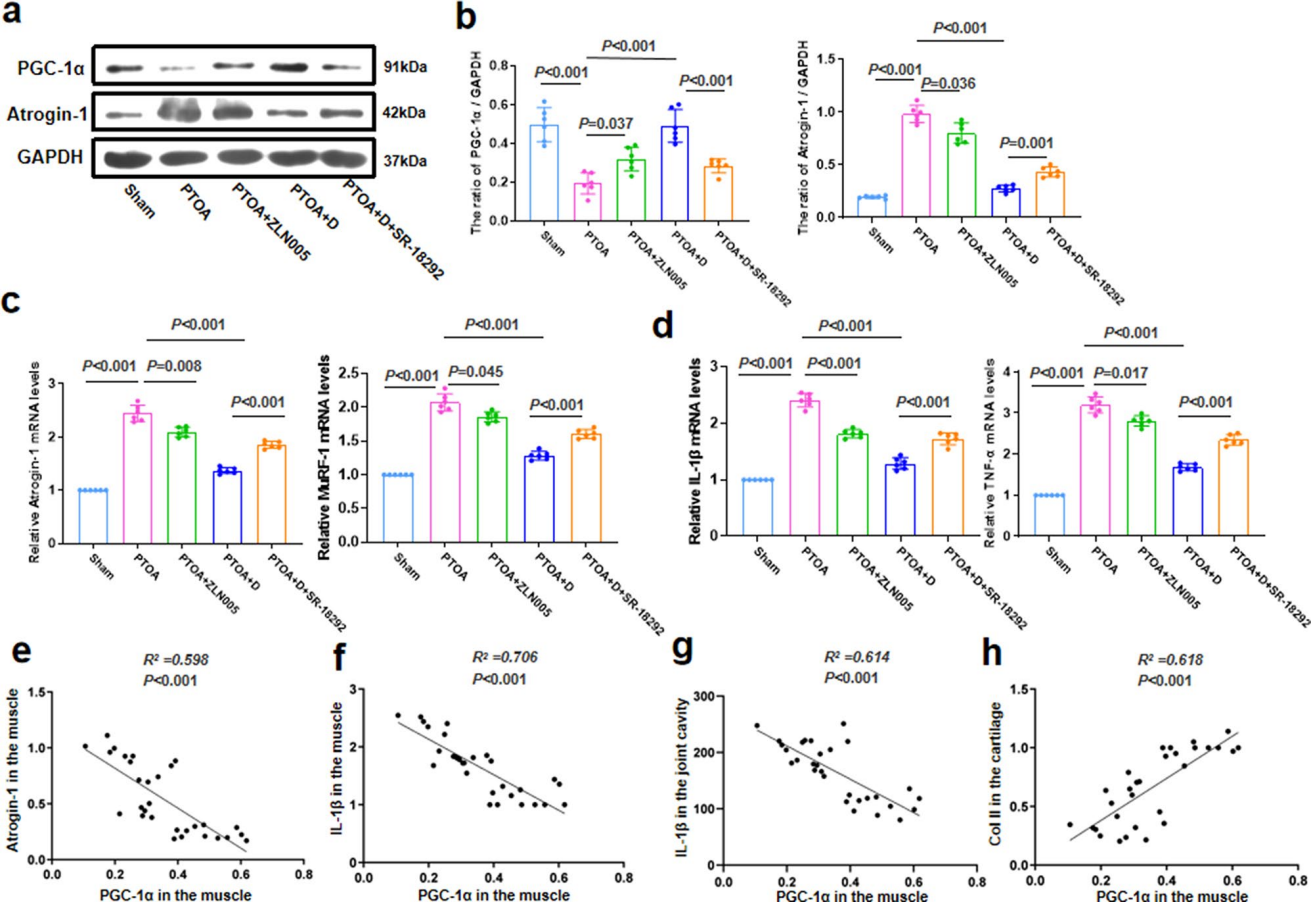
TRE following joint distraction inhibited muscle wasting and inflammation by activating PGC-1α in the PTOA model. **a**-**b** Western blot analysis was conducted to detect and quantify the expression of PGC-1α and Atrogin-1 in the quadriceps muscle. **c-d**. The qRT-PCR was performed for measuring the mRNA expression of *Atrogin-1, MuRF1, IL-1β* and *TNF-α.*** e-h** Linear regression analyses were performed to analyze the associations between PGC-1α in muscle and Atrogin-1, IL-1β in muscle, IL-1β in the joint cavity and Col2a1. *n* = 6

In the rabbit joint instability model of PTOA, PGC-1α activators or inhibitors were used to elucidate the role of TRE following joint distraction in activating PGC-1α in the skeletal muscle. In unstable joints (PTOA group), the expression of PGC-1α in skeletal muscle was downregulated during TRE (Fig. 3a, b). Additionally, the expression of muscle wasting genes (Atrogin-1 and MuRF1) and inflammatory factors (IL-1β and TNF-α) in muscle was increased in the PTOA group (Fig. 3a-d). However, this trend was reversed when the PGC-1α activator (ZLN005) was administered. Notably, TRE following joint distraction (PTOA + D group) not only promoted the expression of PGC-1α in skeletal muscle, but also reduced the expression of muscle wasting genes and inflammatory factors in muscle (Fig. 3a-d). However, this effect was blocked by simultaneous administration of SR-18,292, a PGC-1α inhibitor. In skeletal muscle, the expression of PGC-1α showed a negative correlation with the expression of muscle wasting genes (Atrogin-1) and inflammatory factors (IL-1β) (Fig. 3e-f). Furthermore, the expression of PGC-1α in skeletal muscle was negatively correlated with the expression of inflammatory factors (IL-1β) in the joint cavity (Fig. 3g). However, the expression of PGC-1α in skeletal muscle showed a positive correlation with the expression of Col2a1 in the cartilage (Fig. 3h).

### PGC-1α inhibitors could reduce the effect of TRE following joint distraction in suppressing intra-articular inflammation

**Fig. 4:**
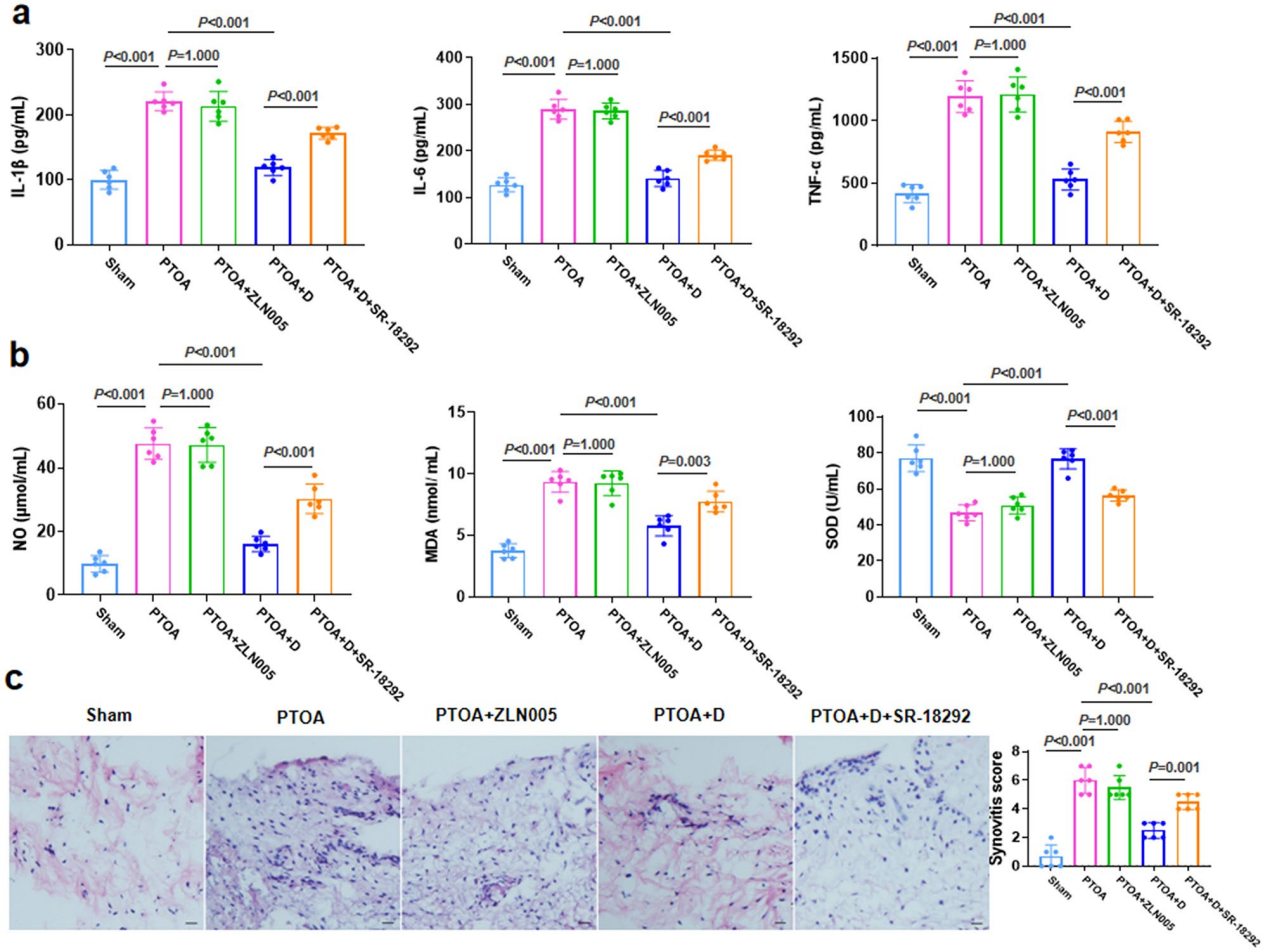
PGC-1α inhibitors can reduce the effect of TRE following joint distraction in suppressing intra-articular inflammation. **a** The expression of IL-1β, IL-6 and TNF-α was detected by ELISA. **b** The expression of NO, MDA and SOD was detected by corresponding assay kits. **c** The synovial tissue was assessed using HE staining and evaluated based on the synovitis score. Scale bar: 50 μm. *n* = 6

Further investigation is needed to examine the impact of PGC-1α supplementation with agonists or inhibitors on intra-articular inflammation during TRE, despite the negative correlation between PGC-1α in skeletal muscle and the expression of inflammatory factors in the joint cavity. The administration of ZLN005 (a PGC-1α activator) in the PTOA group, as depicted in Fig. 4a, did not result in a significant reduction in the expression of IL-1β, IL-6, and TNF-α in the joint cavity. Additionally, it had minimal impact on the expression of NO, MAD, and SOD (Fig. 4b). Similarly, the results of HE staining (Fig. 4c) showed no significant decrease in synovial tissue inflammation in the PTOA + ZLN005 group (Fig. 4c). In the case of joint distraction (PTOA + D group), TRE was found to inhibit intra-articular inflammation and enhance SOD expression in the joint cavity (Fig. 4). After administration of SR-18,292(a PGC-1α inhibitor), intra-articular inflammatory expression increased again, weakening the effect of TRE after joint distraction on inhibiting inflammation (Fig. 4a and c). Furthermore, the results of the synovial inflammation revealed a similar trend. Meanwhile, the expressions of NO and MDA were up-regulated while the expression of SOD was down-regulated (Fig. 4b).

### Inhibitors of PGC-1α weaken the protective effect of cartilage in response to TRE following joint distraction

**Fig. 5:**
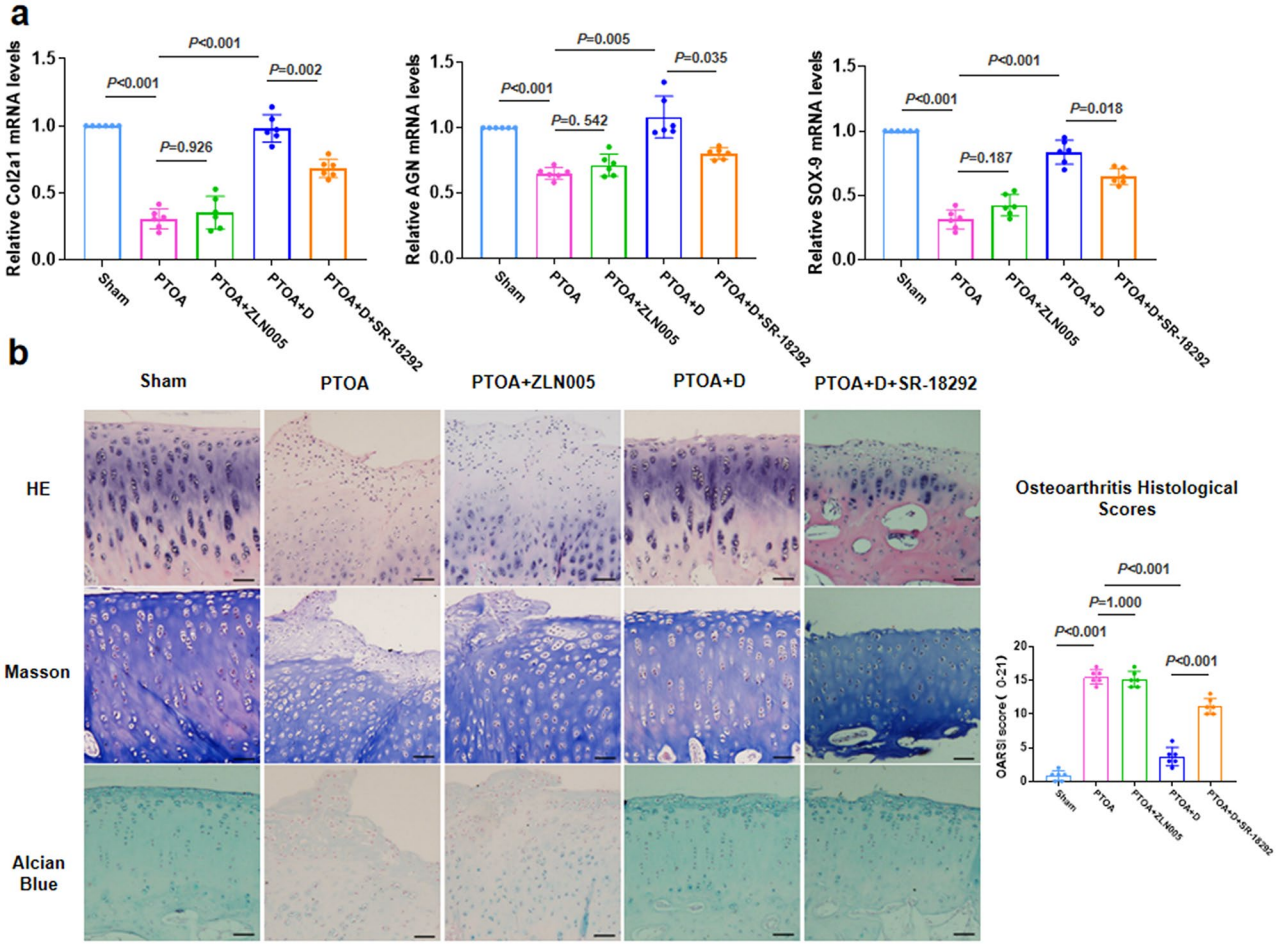
Inhibitors of PGC-1α weaken the protective effect of cartilage in response to TRE following joint distraction. **a** The qRT-PCR was performed for measuring the mRNA expression of *Col2a1, AGN* and *SOX-9*. **b** HE staining, Masson’s trichrome staining, and Alcian blue staining were performed from top to bottom to evaluate the pathological changes in cartilage tissue using the OARSI score. Black scale bar: 50 μm. *n* = 6

Our initial experiments revealed that the combination of TRE and joint distraction exerts a protective effect on cartilage [9]. Figure 5a illustrates the decrease in the expression levels of *Col2a1, AGN* and *SOX-9* upon administration of SR-18,292 (a PGC-1α inhibitor), compared to the PTOA + D group. Additionally, the addition of ZLN005 (a PGC-1α activator) without joint distraction did not enhance the expression levels of *Col2a1, AGN* and *SOX-9*. As described in Fig. 5b, the degeneration and erosion of articular cartilage were more pronounced in the PTOA group and PTOA + ZLN005 group. HE staining revealed that the surface of cartilage tissue in the PTOA + D group appeared smoother and exhibited more distinct layers of chondrocytes. However, chondrocytes in the cartilage tissue treated with SR-18,292 (a PGC-1α inhibitor) exhibited varying degrees of loss and disarray (Fig. 5b). On the surface of cartilage tissue in the PTOA + D + SR-18,292 group, significant loss of collagen fibers stained with Masson’s trichrome and proteoglycans stained with Alcian blue was observed, in addition to the loss of chondrocytes (Fig. 5b). Furthermore, the OARSI score of the PTOA + D group was significantly lower than that of the PTOA + D + SR-18,292 group (Fig. 5b).

### In the premise of joint distraction, TRE could activate PGC-1α and inhibit muscle wasting in skeletal muscles more effectively than NO TRE

**Fig. 6:**
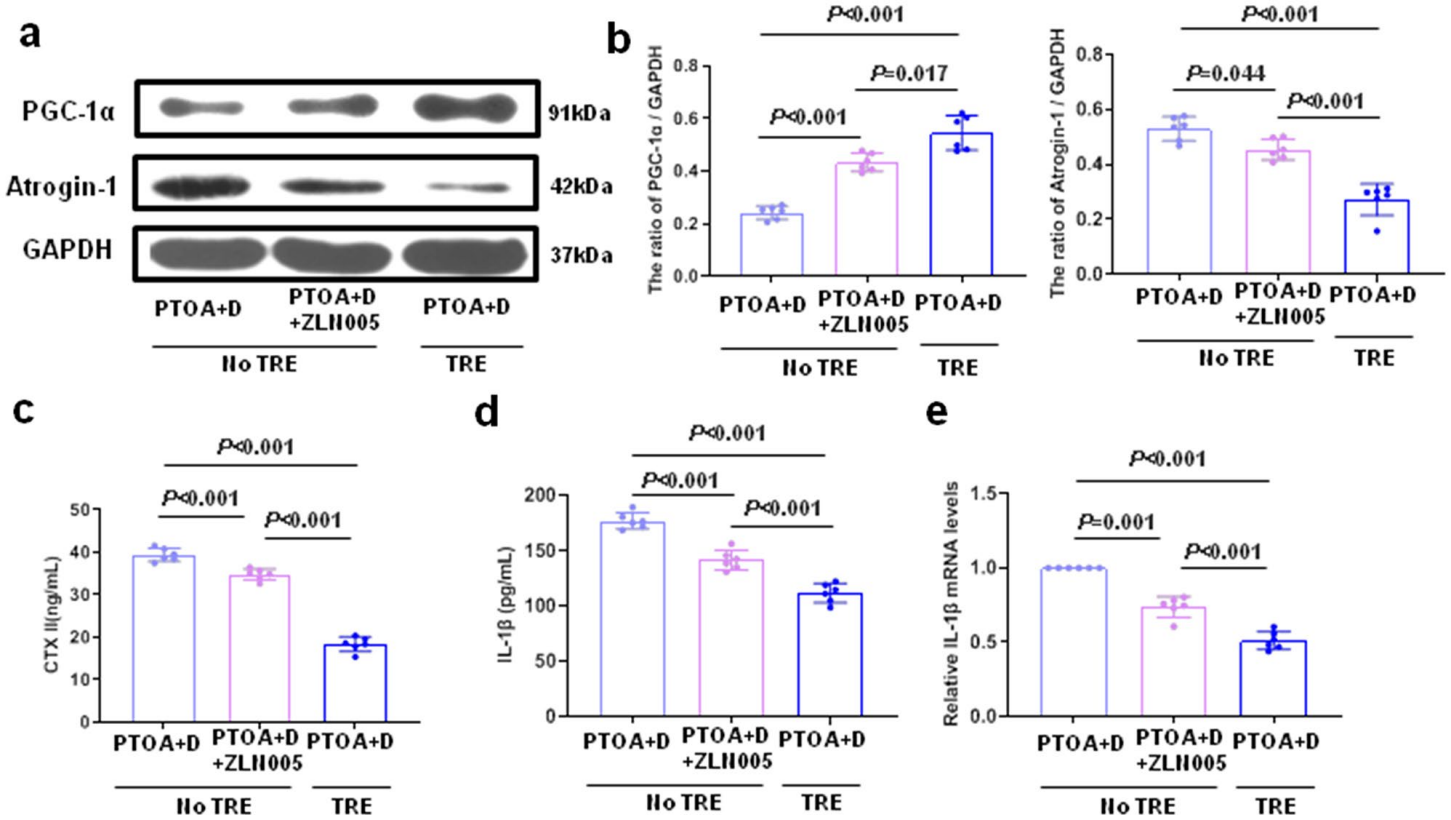
In the premise of joint distraction, TRE could activate PGC-1α and inhibit muscle wasting in skeletal muscles more effectively than NO TRE. **a** CTX II expression in the blood was detected using ELISA. **b** IL-1β expression in the joint cavity was detected using ELISA. **b** The qRT-PCR was performed for measuring the mRNA expression of *IL-1β* in skeletal muscle. **d**-**e** Western blot was performed to detect and quantify the expression of PGC-1α and Atrogin-1 in quadriceps. *n* = 6

The results above suggest that TRE following joint distraction could inhibit intra-articular inflammation and delay PTOA progression by activating PGC-1α in skeletal muscle. Therefore, in the premise of joint distraction, we conducted further investigation into the impact of TRE or No TRE on PGC-1α in skeletal muscle. As shown in Fig. 6a and b, the results of the western blot analysis demonstrated that TRE following joint distraction upregulated the expression of PGC-1α in skeletal muscle and downregulated the expression of Atrogin-1 in skeletal muscle compared to the No TRE group. CTX II levels in the blood were frequently utilized for evaluating cartilage degeneration. According to the expression level of CTX II, it was significantly lower in the TRE group than in the No TRE group. In the No TRE group, ZLN005, a PGC-1α activator, was administered to decrease CTX II expression in the case of joint distraction. (Fig. 6c). Consistent with the above result, the results of IL-1β in the joint cavity and *IL-1β* in skeletal muscle revealed similar trends (Fig. 6d, e).

## Discussion

Unlike primary OA, PTOA is characterized by a clear history of joint trauma. After joint trauma, early chondrocyte necrosis triggers inflammatory reactions within the joint, which worsen the breakdown of the cartilage matrix and ultimately result in PTOA [18]. After joint trauma, patients may require immobilization or surgery, and long-term recovery often involves significant muscle wasting [19]. While muscle wasting occurs in most patients following joint trauma, not all of them develop PTOA. Nonetheless, muscle wasting can exacerbate joint instability in OA patients, leading to increased joint pain [19]. On the other hand, the progression of OA can lead to muscle inflammation and redox disorders, which ultimately leads to muscle wasting [11]. Additionally, as the severity of knee OA increases in women, there is a tendency for the muscle mass of the lower limbs to decline [20]. However, in male mice, over-expression of PGC-1α in skeletal muscle may help withstand muscle fatigue and prevent muscle wasting [21]. In our study, we found that the decreased expression of PGC-1α in the quadriceps muscle in patients with OA is correlated with the severity of OA. Furthermore, the activation of PGC-1α is crucial in preventing muscle wasting caused by oxidative damage or inflammation [22]. PGC-1α serves as the primary regulator of mitochondrial biosynthesis, and current research suggests its involvement in the development of PTOA through mitochondria [4]. Therefore, the activation of PGC-1α not only improves muscle wasting but also has the potential to delay PTOA progression. Considering that exercise activates PGC-1α in skeletal muscle [14], it is reasonable to assume that joint distraction combined with TRE for the prevention of PTOA may be associated with this activation. Therefore, we measure the expression of PGC-1α in skeletal muscle after TRE. Our results indicate that TRE following joint distraction activates the expression of PGC-1α in skeletal muscle and reduces intra-articular inflammation, leading to a delay in PTOA progression. However, these effects can be attenuated by the PGC-1α inhibitor (SR-18,292).

Joint instability causes excessive mechanical stress on the articular cartilage, leading to early degeneration and necrosis, which further aggravates intra-articular inflammation and cartilage degeneration [23]. It is important to note that the mechanical injury to cartilage caused by joint instability will worsen during exercise [24]. Our previous findings are consistent with the above results, demonstrating that without joint distraction, TRE leads to increased levels of intra-articular pro-inflammatory factors (such as IL-1β, TNF-α, and IL-6), worsened articular surface wear, and reduced expression of cartilage regeneration genes in the PTOA group [9]. Under the same TRE conditions, the PTOA + D group exhibited lower levels of intra-articular pro-inflammatory factors and synovial tissue inflammation, reduced articular surface wear, and increased expression of cartilage regeneration genes compared to the PTOA group. Additionally, mechanical cartilage injury leads to chondrocyte damage and death, resulting in an increased release of pro-inflammatory factors (such as IL-1β and TNF-α), further exacerbating cartilage damage [25]. Intramuscular pro-inflammatory factors can induce muscle wasting through various mechanisms, including the disruption of muscle protein synthesis and catabolism balance, as well as the acceleration of cellular aging [11, 26]. Muscle atrophy gene 1 (Atrogin-1; also known as MAFbx) and muscle ring finger 1 (MuRF1) are muscle-specific ubiquitin ligases that play a crucial role in muscle wasting [27]. In this experiment, TRE following joint distraction significantly suppressed the expression of Atrogin-1 and MuRF1 in muscle tissue compared to the PTOA group. Cunha et al. demonstrated that the upregulation of Atrogin-1 and MuRF1 expression in the quadriceps muscle of rats with ACLT-induced OA is linked to an inflammatory response [28]. Our results showed that TRE following joint distraction effectively decreased the expressions of IL-1β and TNF-α in skeletal muscle. Therefore, following joint distraction, TRE alleviates both intra-articular and muscle inflammation, leading to the suppression of muscle atrophy.

Exercise has the potential to enhance quadriceps muscle strength and alleviate pain in individuals with OA [29]. Additionally, exercise stimulates the activation of PGC-1α in skeletal muscle, leading to enhanced mitochondrial function and reduction in muscle wasting [14, 30]. In muscle tissue affected by atrophy, there is a decrease in the expression of SOD, the primary antioxidant enzyme responsible for eliminating superoxide, while the expression of MDA is elevated [31]. In our study, we found that TRE following joint distraction enhances the expression of SOD in the joint, while simultaneously decreasing the expression of MDA. Nonetheless, PGC-1α inhibitors have the potential to reverse this trend. Exercise induces the activation of skeletal muscle PGC-1α, enhancing mitochondrial function and exerting antioxidant and anti-inflammatory effects [14, 15], ultimately reducing muscle inflammation and improving muscle wasting. Our results indicate that the expression of skeletal muscle PGC-1α is negatively correlated with the expression of IL-1β in both the muscle and the joint cavity. PGC-1α inhibitors not only increase muscle inflammation and wasting but also alleviate the inhibitory effects of TRE following joint distraction on joint inflammation. The strong correlation between skeletal muscle inflammation, muscle wasting, and intra-articular inflammation suggests that PGC-1α in skeletal muscle may mitigate intra-articular inflammation by suppressing muscle inflammation and muscle wasting. However, further research is required to elucidate the specific mechanisms. Furthermore, exercise-induced activation of PGC-1α exerts anti-inflammatory effects, leading to a reduction in the expression of age-related inflammatory factors, such as TNF-α [32]. Our results showed that joint distraction combined with PGC-1α agonists (ZLN005) resulted in a reduction in intra-articular and muscle inflammation, as well as a decrease in the expression of Atrogin-1. However, this effect was not as pronounced as that observed with joint distraction combined with TRE. Both aerobic exercise and resistance exercise suppressed the expression of Atrogin-1 and MuRF1, regulated myogenesis, and mitigated protein degradation in mice, ultimately alleviating skeletal muscle wasting [33]. In unstable joints, our findings suggest that while the combination of TRE and PGC-1α activators can enhance the expression of PGC-1α in skeletal muscle, it does not effectively suppress muscle inflammation and wasting, nor does it inhibit intra-articular inflammation or alleviate PTOA progression. This is due to the fact that exercise in unstable joints can cause increased mechanical damage to cartilage, trigger intra-articular inflammation [23, 24], and consequently result in muscle inflammation and wasting [11]. Therefore, TRE following joint distraction has the potential to suppress intra-articular and muscle inflammation through the activation of PGC-1α in skeletal muscle, consequently enhancing muscle wasting and delaying the progression of PTOA.

Although TRE following joint distraction may potentially delay the progression of OA by enhancing PGC-1α in skeletal muscle, our study has certain limitations. Firstly, further research is needed to elucidate the specific mechanism by which PGC-1α in skeletal muscles inhibits intra-articular inflammation. Additionally, there is still room for improvement in the hinged external fixator.

## Conclusions

The reduced expression of PGC-1α in the quadriceps femoris of patients with OA is associated with the severity of OA. In the rabbit model of joint instability with PTOA, joint distraction serves as a precondition for TRE to activate PGC-1α in skeletal muscle. Our results demonstrated TRE after joint distraction may activate PGC-1α in skeletal muscle to resist skeletal muscle and intra-articular inflammation in PTOA progression, thereby improving muscle wasting and promoting cartilage regeneration, ultimately delaying the progression of PTOA. Therefore, this study offers valuable insights into the potential therapeutic benefits of this intervention in preventing muscle wasting and delaying the progression of PTOA.

## Data Availability

The data sets generated and analyzed during the current study are available from the corresponding author on reasonable request.
